# Unexpected Spontaneous Consolidation of a Chronic Scaphoid Nonunion: A Report of a Rare Case and Review of Previously Published Cases

**DOI:** 10.7759/cureus.111610

**Published:** 2026-06-27

**Authors:** Taieb Iheb, Khairi Saibi, Doha Laamarti, Lilya Sellami, Mouna Ounaies

**Affiliations:** 1 Department of Hand Surgery, Mohamed Kassab Institute of Orthopedics, Tunis, TUN; 2 Department of Plastic and Hand Surgery, University of Tunis, Tunis, TUN

**Keywords:** avascular necrosis, scaphoid, scaphoid fracture, scaphoid nonunion, spontaneous union

## Abstract

Scaphoid nonunion is a common complication of missed or inadequately treated scaphoid fractures and is generally considered an indication for surgical treatment because of the risk of progressive carpal collapse and degenerative arthritis. Spontaneous union of an established scaphoid nonunion is exceptionally rare, particularly in the presence of radiological findings suggestive of proximal pole vascular compromise. We report the case of a 23-year-old right-handed manual worker with a chronic scaphoid nonunion. The patient sustained an initial wrist injury in 2017 that was treated conservatively after normal radiographs and was subsequently lost to follow-up. Following a new wrist trauma in 2023, imaging revealed a stage IIA scaphoid nonunion according to the Alnot classification. Computed tomography demonstrated a persistent fracture line with sclerotic margins and increased density of the proximal fragment, suggestive of avascular compromise. Surgical reconstruction using a vascularized bone graft according to the Zaidemberg technique was planned. However, radiographs obtained 13 months after the diagnosis and immediately before surgery unexpectedly demonstrated complete union of the scaphoid. Computed tomography confirmed trabecular bridging and complete consolidation, leading to cancellation of the planned procedure. Spontaneous healing of an established scaphoid nonunion has been reported only rarely in the literature. Although the mechanisms remain poorly understood, progressive revascularization, favorable biological healing potential, and intrinsic mechanical stability have been proposed as possible explanations. This case highlights the exceptional possibility of spontaneous union in chronic scaphoid nonunion, even in the presence of adverse radiological features, and emphasizes the importance of repeat imaging before undertaking surgical treatment.

## Introduction

Scaphoid fractures are the most common carpal fractures, at a range of 70% [1]. Nonunion occurs in approximately 5-15% of cases, particularly after delayed diagnosis or inadequate treatment [2]. Owing to its unique retrograde vascular supply, the proximal pole is especially vulnerable to vascular compromise, which may impair healing and increase the risk of nonunion [3].

The natural history of untreated scaphoid nonunion is generally unfavorable, potentially leading to progressive carpal instability, chronic pain, and degenerative arthritis [4]. Consequently, surgical treatment with internal fixation and bone grafting remains the standard management for established nonunion [5]. Although uncommon, spontaneous consolidation of chronic scaphoid nonunion has been described in the literature, with only a limited number of documented cases available.

We present a case of spontaneous consolidation of a chronic scaphoid nonunion despite radiographic and CT findings suggestive of compromised proximal pole biology.

## Case presentation

A 23-year-old right-handed manual worker with no significant medical history except for smoking (estimated at two pack-years) presented with a history of two wrist injuries.

The initial trauma occurred in August 2017 following a fall onto an outstretched hand. Initial radiographs did not reveal any fracture, and the patient received conservative treatment before being lost to follow-up. No clinical or radiological evaluations were performed during the subsequent six-year interval.

In August 2023, he sustained a second wrist injury following the same mechanism. At presentation, the patient reported mild chronic wrist pain exacerbated by manual activities. Clinical examination revealed mild limitation of wrist motion, tenderness over the anatomical snuffbox and scaphoid tubercle, and a positive scaphoid compression test. Grip strength was clinically decreased compared with the contralateral side; however, objective dynamometric assessment was not available due to the unavailability of a Jamar dynamometer. No signs of carpal instability or neurovascular deficit were identified. The QuickDASH score [6] was 30 (Table 1).

**Table 1:** Comparative clinical assessment at initial presentation and after spontaneous healing of the scaphoid nonunion

Parameter	Initial presentation	13-month follow-up
Wrist flexion (°)	40	60
Wrist extension (°)	50	70
Ulnar deviation (°)	25	25
Radial deviation (°)	10	20
Forearm pronation (°)	80	80
Forearm supination (°)	80	80
Pain (VAS, 0–10)	2/10	1/10
Grip strength	40% of the contralateral side	Clinically improved (dynamometer not available)
QuickDASH	30	10
Neurovascular deficits	No	No
Watson’s shift test	Negative	Negative

Due to logistical and administrative constraints, including delays in scheduling the pre-anesthetic assessment and in securing operating room availability, the planned surgical procedure was postponed for 13 months. During this interval, no additional clinical or radiological evaluation was performed.

At the preoperative reassessment, 13 months after the initial diagnosis of chronic scaphoid nonunion, the patient reported substantial clinical improvement with minimal residual pain (VAS 1/10). Wrist flexion improved from 40° to 60°, extension from 50° to 70°, and radial deviation from 10° to 20°. The QuickDASH score improved from 30 to 10. Grip strength was clinically improved compared with the initial presentation, and the patient had returned to his previous manual occupation without restrictions (Table 1).

Imaging findings

Initial wrist radiographs demonstrated a stage IIA scaphoid nonunion according to the Alnot classification and IIA according to the Bahri classification [5,7]. This stage corresponds to a stable nonunion without carpal instability.

**Figure 1:**
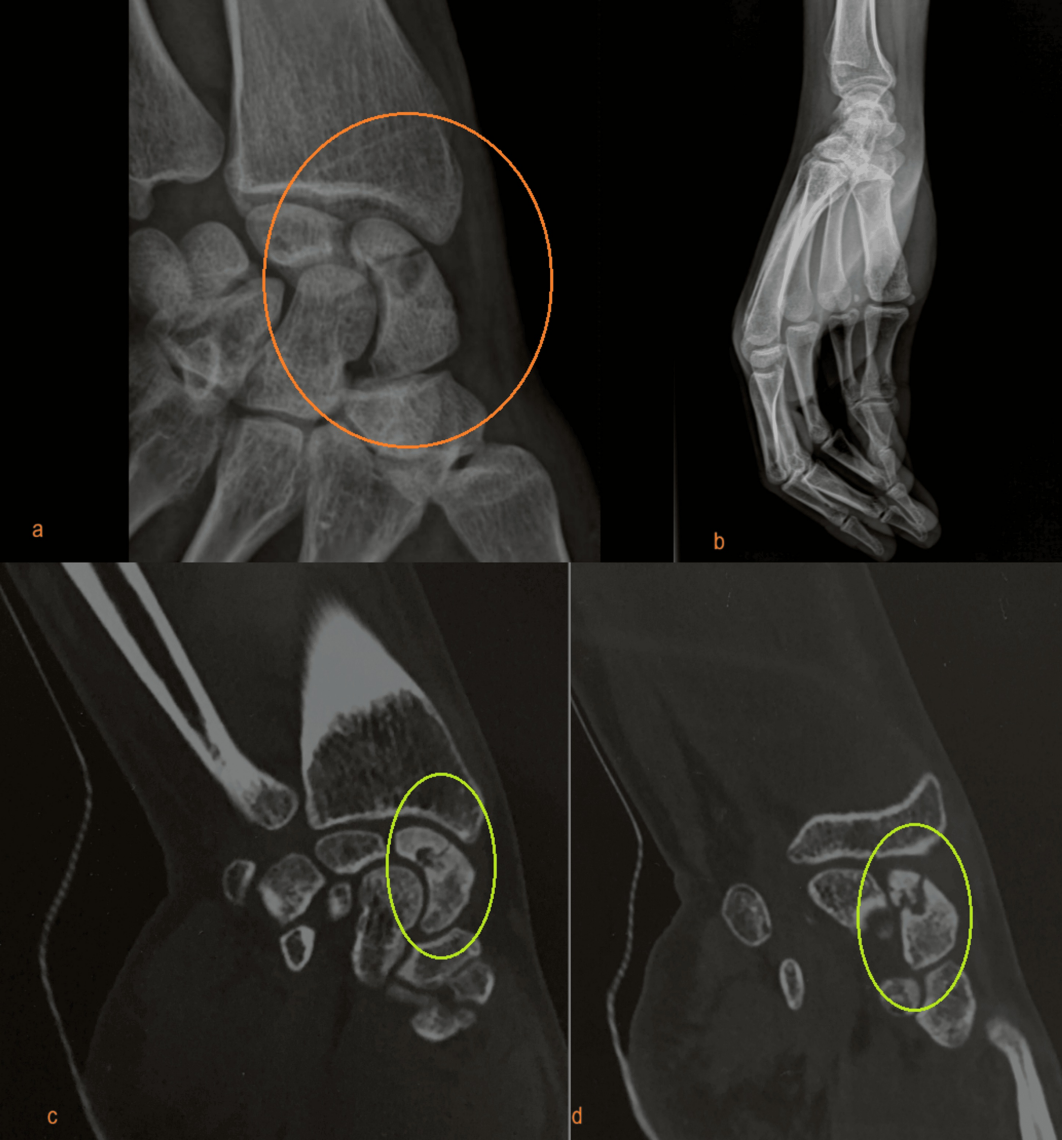
Radiographs and CT scan showing a stage IIA scaphoid nonunion with proximal pole sclerosis suggestive of avascular compromise (a) Posteroanterior and (b) lateral wrist radiographs demonstrating a stage IIA scaphoid nonunion according to the Alnot and Bahri classifications (orange circle). CT scans (c, d) showing a persistent fracture line with sclerotic margins and increased density of the proximal fragment, suggestive of avascular compromise.

CT confirmed the absence of union, showing a persistent fracture line with sclerotic margins and increased density of the proximal fragment, suggesting compromised proximal pole biology (Figure 1). No DISI (dorsal intercalated segment instability) deformity, humpback deformity, or carpal malalignment was identified.

Given the chronic nature of the lesion and the radiological findings, surgical reconstruction using a vascularized bone graft according to the Zaidemberg technique [8] was planned.

Unexpectedly, radiographs obtained before the scheduled surgery, 13 months after the diagnosis of scaphoid nonunion, demonstrated complete osseous consolidation. CT confirmed trabecular bridging and complete union across the previous nonunion site. Magnetic resonance imaging (MRI) was not performed; therefore, the vascular status of the proximal pole could not be definitively assessed, and avascular compromise remained a radiological suspicion based on CT findings alone.

**Figure 2:**
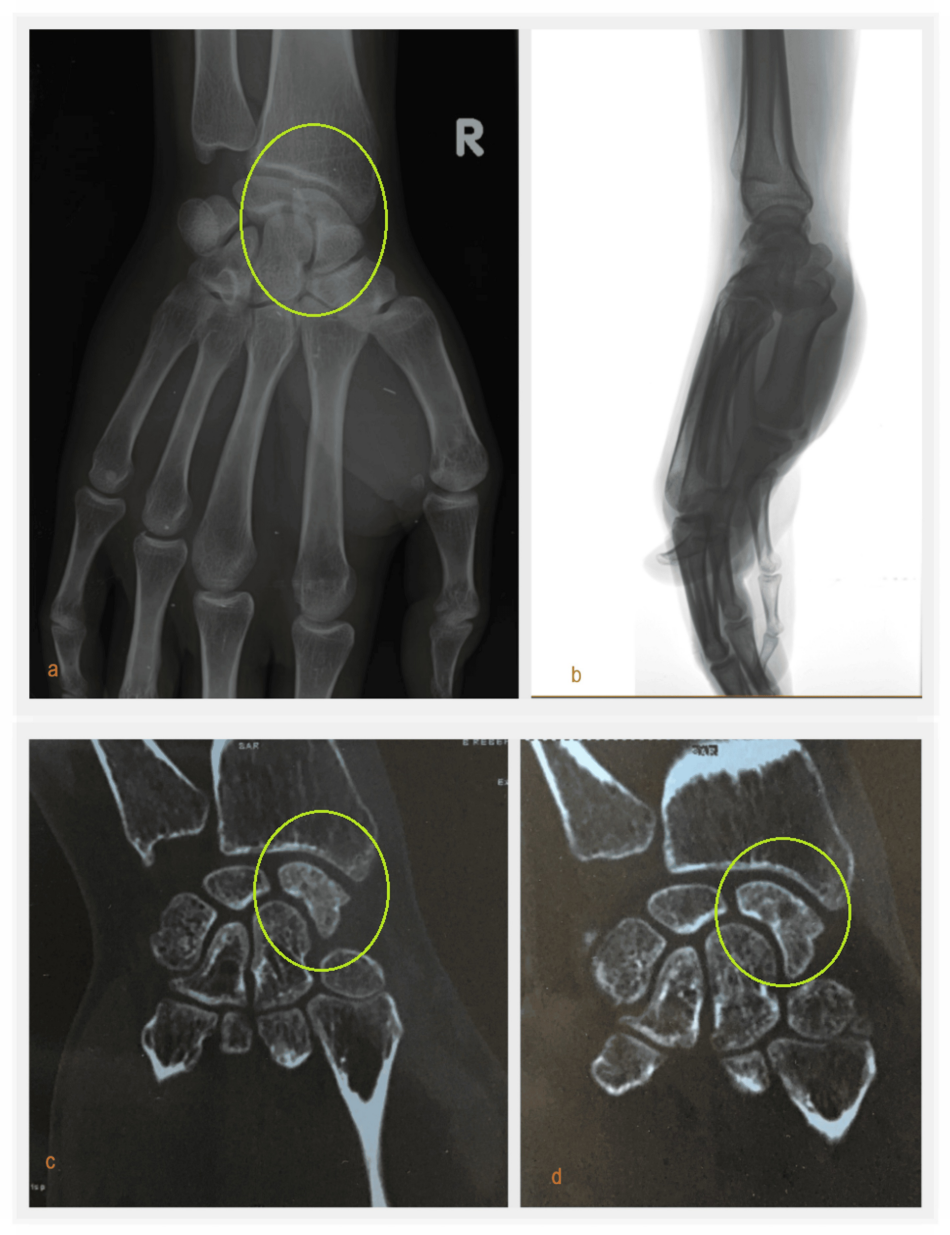
Imaging at follow-up demonstrating complete spontaneous union of the scaphoid, with CT confirmation of trabecular bridging and complete consolidation of the previous nonunion (a–d) Trabecular bridging and complete consolidation of the previous nonunion (green circle).

Consequently, in view of the confirmed union and clinical improvement, surgical intervention was considered unnecessary, and the planned procedure was cancelled (Figure 2).

## Discussion

Scaphoid nonunion occurs in approximately 5-15% of scaphoid fractures [2] and remains a challenging condition because of the unique retrograde blood supply of the scaphoid, particularly to the proximal pole, which is especially vulnerable to vascular compromise and impaired healing. Several risk factors for nonunion have been identified, including fracture displacement, proximal pole involvement, delayed diagnosis, inadequate immobilization, and avascular necrosis. Consequently, surgical intervention is generally recommended, especially in the presence of longstanding nonunion or radiological signs suggestive of compromised vascularity [3].

Spontaneous union of an established scaphoid nonunion is an exceptionally uncommon event. To our knowledge, only two well-documented cases of spontaneous healing of established scaphoid nonunion have been reported previously. Roolker et al. [9] first described spontaneous healing of a scaphoid nonunion in 1998, while Park et al. subsequently reported spontaneous union of a proximal pole nonunion despite the traditionally poor healing potential associated with this location [10].

These rare observations challenge the conventional assumption that established scaphoid nonunion invariably requires surgical treatment and suggest that healing potential may persist in selected cases.

The biological mechanisms underlying spontaneous union remain poorly understood. Progressive revascularization of the nonunion site appears to be the most plausible explanation. Cheema and Cheema [3] emphasized the critical role of vascularity in scaphoid healing and highlighted that the vascular status of the proximal fragment is dynamic rather than static, potentially allowing restoration of osteogenic activity over time. Mechanical stability at the nonunion site may represent another contributing factor. Since instability and fracture displacement are recognized risk factors for persistent nonunion, residual stability provided by fibrous tissue bridging the fracture gap may create a favorable biological environment for gradual osseous consolidation [2].

In addition, biological factors intrinsic to the patient may have contributed to the observed healing. Bone morphogenetic proteins (BMPs) and other growth factors play a central role in osteogenesis and fracture repair. In their systematic review, Polmear et al. highlighted the importance of BMP-mediated biological activity in the treatment of scaphoid nonunion, suggesting that variations in local osteogenic potential and bone remodeling capacity may influence healing outcomes [11]. Although direct evidence is lacking in the present case, enhanced endogenous osteogenic activity may represent one possible explanation for the rare occurrence of spontaneous union in selected patients. Similarly, physiological loading during daily activities may have stimulated bone formation through mechanobiological pathways, promoting progressive trabecular bridging across the nonunion site. However, the involvement of BMP activity and mechanobiological stimuli remains speculative and should be regarded as biologically plausible hypotheses rather than proven mechanisms. As no biological, histological, or advanced imaging investigations were performed, the exact factors responsible for consolidation in this patient cannot be determined with certainty.

Our case is particularly remarkable because spontaneous consolidation occurred despite the chronic nature of the lesion and CT findings suggestive of proximal fragment vascular compromise. Such radiological features are generally considered unfavorable prognostic factors and frequently constitute an indication for vascularized bone grafting. Similar to the case reported by Park et al., spontaneous healing occurred despite presumed biological impairment of the proximal fragment. However, unlike previously reported cases, complete union in our patient was documented by CT immediately before a planned Zaidemberg vascularized bone graft procedure, providing objective evidence of spontaneous consolidation. This observation further expands the limited body of evidence on spontaneous healing of established scaphoid nonunion and underscores the incomplete understanding of the biological mechanisms governing scaphoid healing (Table 2).

**Table 2:** Comparison of reported cases of spontaneous healing of scaphoid nonunion

Characteristic	Roolker et al. (1998) [9]	Park et al. (2012) [10]	Present Case
Patient age	19 years	23 years	23 years
Sex	Male	Male	Male
Initial injury	Scaphoid fracture	Scaphoid fracture	Wrist trauma in 2017, fracture initially missed
Nonunion location	Scaphoid nonunion	Proximal pole scaphoid nonunion	Scaphoid nonunion with proximal pole involvement
Time from injury to diagnosis	10 months	6 months	Approximately 6 years
Clinical symptoms	Persistent wrist pain	Mild wrist symptoms	Mild pain and moderate limitation of wrist motion
Imaging findings	Established scaphoid nonunion	Proximal pole nonunion	Alnot stage IIA nonunion with sclerotic margins and CT findings suggestive of proximal pole avascular compromise
Evidence of avascular necrosis	Not reported	Not reported	Suspected on CT scan
Planned treatment	Surgical treatment considered	Surgical treatment discussed	Vascularized bone graft (Zaidemberg technique) scheduled
Time to spontaneous union	18 months	2 years	13 months after diagnosis
Confirmation of union	Radiographs + scintigraphy	Radiographs + MRI	Radiographs and CT scan
Surgery performed	No	No	No (cancelled after documented union)
Outcome	Complete spontaneous union	Complete spontaneous union	Complete spontaneous union
Particularity of the case	First reported case in the literature	Proximal pole nonunion with spontaneous healing	Chronic nonunion with suspected proximal pole vascular compromise and CT-confirmed spontaneous union

The main limitation of this report is the absence of MRI evaluation, which prevented definitive assessment of proximal pole vascularity. Nevertheless, CT findings suggested vascular compromise and complete union was objectively confirmed by computed tomography.

## Conclusions

This case highlights the rare possibility of spontaneous consolidation of an established scaphoid nonunion, even in the presence of radiological features traditionally considered unfavorable. Possible contributing factors may include the absence of carpal instability, preserved mechanical conditions, progressive revascularization, and intrinsic biological healing potential. However, these mechanisms remain speculative and cannot be confirmed from a single case.

Although this observation does not modify current treatment recommendations for chronic scaphoid nonunion, it emphasizes the potential for unexpected healing in selected cases. Repeat imaging before definitive surgical intervention may be considered in patients with clinical improvement or in those with prolonged delays before surgery. However, this isolated case does not support routine reassessment for all chronic scaphoid nonunions. Further reports are needed to better understand the biological factors that may contribute to this uncommon phenomenon.
